# CRISPR/Cas-based CAR-T cells: production and application

**DOI:** 10.1186/s40364-024-00602-z

**Published:** 2024-05-31

**Authors:** Ping Song, Qiqi Zhang, Zhiyong Xu, Yueli Shi, Ruirui Jing, Dingcun Luo

**Affiliations:** 1 Department of Surgical Oncology, Affiliated Hangzhou First People’s Hospital, Westlake University School of Medicine, No. 261, Huansha Road, Shangcheng district, Hangzhou 310006, Zhejiang P. R. China; 2 Bone Marrow Transplantation Center, the First Affiliated Hospital, Zhejiang University School of Medicine, Hangzhou, China; 3 Department of Respiratory Medicine, The Fourth Affiliated Hospital, International Institutes of Medicine, Zhejiang University School of Medicine, Yiwu City, China; 4 The Fourth Clinical Medical College, Zhejiang Chinese Medical University, Hangzhou 310006, Zhejiang China

**Keywords:** Immunotherapy, CAR-T cell therapy, Gene editing, CRISPR/Cas9

## Abstract

Chimeric antigen receptor T cell (CAR-T) therapy has revolutionized the treatment approach for cancer, autoimmune disease, and heart disease. The integration of CAR into T cells is typically facilitated by retroviral or lentiviral vectors. However, the random insertion of CARs can lead to issues like clonal expansion, oncogenic transformation, variegated transgene expression, and transcriptional silencing. The advent of precise gene editing technology, like Clustered Regularly Interspaced Short Palindromic Repeats (CRISPR), allows for controlled and precise genome modification, facilitating the translation of CAR-T research to the clinical applications. This review aims to provide a comprehensive analysis of the application of CRISPR gene editing techniques in the context of precise deletion and insertion methodologies, with a specific focus on their potential for enhancing the development and utilization of CAR-T cell therapy.

## Introduction

### Overview of CAR-T cell therapy

Chimeric antigen receptor T cell (CAR-T) cell therapy has demonstrated remarkable efficacy and safety for the treatment of hematological malignancies in recent years. CAR constructs, which consist of an extracellular antigen-binding domain (single-chain fragment variable, scFv), transmembrane hinges, and intracellular signal domains (such as CD3ζ chain and costimulatory domain), enable CAR-T cells to specifically identify, activate, and eradicate tumor cells in an antigen-specific and MHC-independent manner [1]. So far, six CAR-T products leveraging this mechanism have been approved for the therapeutic management of B-cell acute lymphoblastic leukemia/Non-Hodgkin Lymphoma (B-ALL/NHL) or Multiple Myeloma (MM). However, the production of all six products involves the introduction of CAR genes into human primary T cells through infection with lentivirus (LV) or retroviral vector (RV). Consequently, this integration process may result in clone amplification, carcinogenic transformation, mutated transgenic expression, and transcriptional silencing. Additionally, CAR-T cell exhaustion, toxicity concerns, and limited autologous cell availability have hindered widespread adoption.

### Briefs of gene editing technologies

Gene editing technologies play a crucial role in the production and optimization of CAR-T cells for anti-tumor purposes. These technologies, including transcription activator-like effector nucleases (TALENs), zinc-finger nucleases (ZFNs), and clustered regularly interspaced short palindromic repeats (CRISPR), facilitate precise modification and manipulation of genes in CAR-T cell engineering [2].

ZFNs and TALENs are chimeric nucleases comprising a modular DNA-binding domain and a sequence-independent cleavage domain derived from the FokΙ restriction enzyme [3]. Utilizing a zinc finger protein or transcriptional activator-like effect (TALE) domain, they recognize and bind to DNA at a specific sequence. Subsequently, they introduce an endonuclease to cleave the sequence, resulting in a DNA double-stranded break (DSB) at the targeted locus. However, the broad application of TALENs and ZFNs is hindered by the time-consuming and complex process involved, as a specific editing protein is required for each version of genome editing [4, 5]. Following the DSB, the eukaryotic cellular DNA repair system repairs the DSBs through either the homology-directed repair (HDR) or non-homologous end joining (NHEJ) pathways [6], leading to targeted integration or disruption of genes, depending on the pathway utilized.

In contrast, the CRISPR/Cas system is widely recognized as a powerful gene editing tool due to its simple design and high efficiency, offering promising prospects for cancer treatment. CRISPR/Cas system has greatly simplified the gene editing process and is now extensively applied in cell therapy, with recent progress toward clinical applications. Initially reported in *E. coli*, CRISPRs were later identified as an intrinsic adaptive immune system in eukaryotic cells, providing defense against foreign DNA. Currently, the most used systems are CRISPR/Cas9 and CRISPR/Cas12a. The CRISPR/Cas9 technology involves a 20-base pair single guide RNA (sgRNA) that guides the DNA endonuclease to the desired cutting site. This site is specified by a protospacer adjacent motif (PAM) sequence located downstream of the cleavage site within the target DNA [7]. On the other hand, the CRISPR/Cas12a system recognizes the TTTV sequence on the genome and requires only a single crRNA to cut the genomic DNA. This process produces sticky ends that are repaired similarly to CRISPR/Cas9. MEGA-CRISPR harnesses Cas13d’s RNA-directed editing capabilities through tailored guide RNA (gRNA) design, enabling precise recognition and cleavage of target RNA sequences for editing [8]. Here is a comparison of the advantages and drawbacks of CRISPR/Cas9, CRISPR/Cas12a, and CRISPR/Cas13d in CAR-T therapy, presented in Table 1. These characteristics help better understand the strengths and limitations of each system in the context of CAR-T therapy. The CRISPR/dCas9 system is utilized to modulate transcriptional activities by recruiting transcriptional activators or repressors to specific loci, known as CRISPR activation (CRISPRa) and CRISPR interference (CRISPRi), respectively. Provided below is an in-depth exploration regarding the generation of CAR-T cell therapy leveraging the aforementioned gene editing approaches [9].

**Table 1:** Key features of CRISPR/Cas9, CRISPR/Cas12a, and CRISPR/Cas13d in CAR-T therapy

Feature	CRISPR/Cas9	CRISPR/Cas12a	CRISPR/Cas13d
Target gene editing efficiency	High	Moderate to high	Low
PAM sequence requirements	NGG	TTTN	N/A (targets RNA)
Gene editing precision	High	Moderate	High
Applicability to large-scale genome editing	Yes	Yes	No
Suitability for point mutations and insertions/deletions	Yes	Yes	Yes
Targeting capability for RNA and DNA	DNA and RNA	DNA	RNA
Type of target modified	Genomic DNA	Genomic DNA	RNA
Structural complexity	Larger	Smaller	Moderate
Therapeutic potential	High	Moderate	Moderate to low
Design flexibility	High	Moderate	High
Economic practicality	High	High	Low

## The production of CRISPR/Cas-based CAR-T cells

Currently, there are three primary approaches for generating CAR-T cells utilizing the CRISPR system, with the most conventional being the CRISPR/Cas9 system, alongside CRISPR/Cas12a and CRISPR/Cas13d. The procedures for generating CAR-T cells utilizing these systems will be elaborated upon in the following sections.

### Current state of research on the production of CAR-T cells using the CRISPR/Cas9 system

**Fig. 1:**
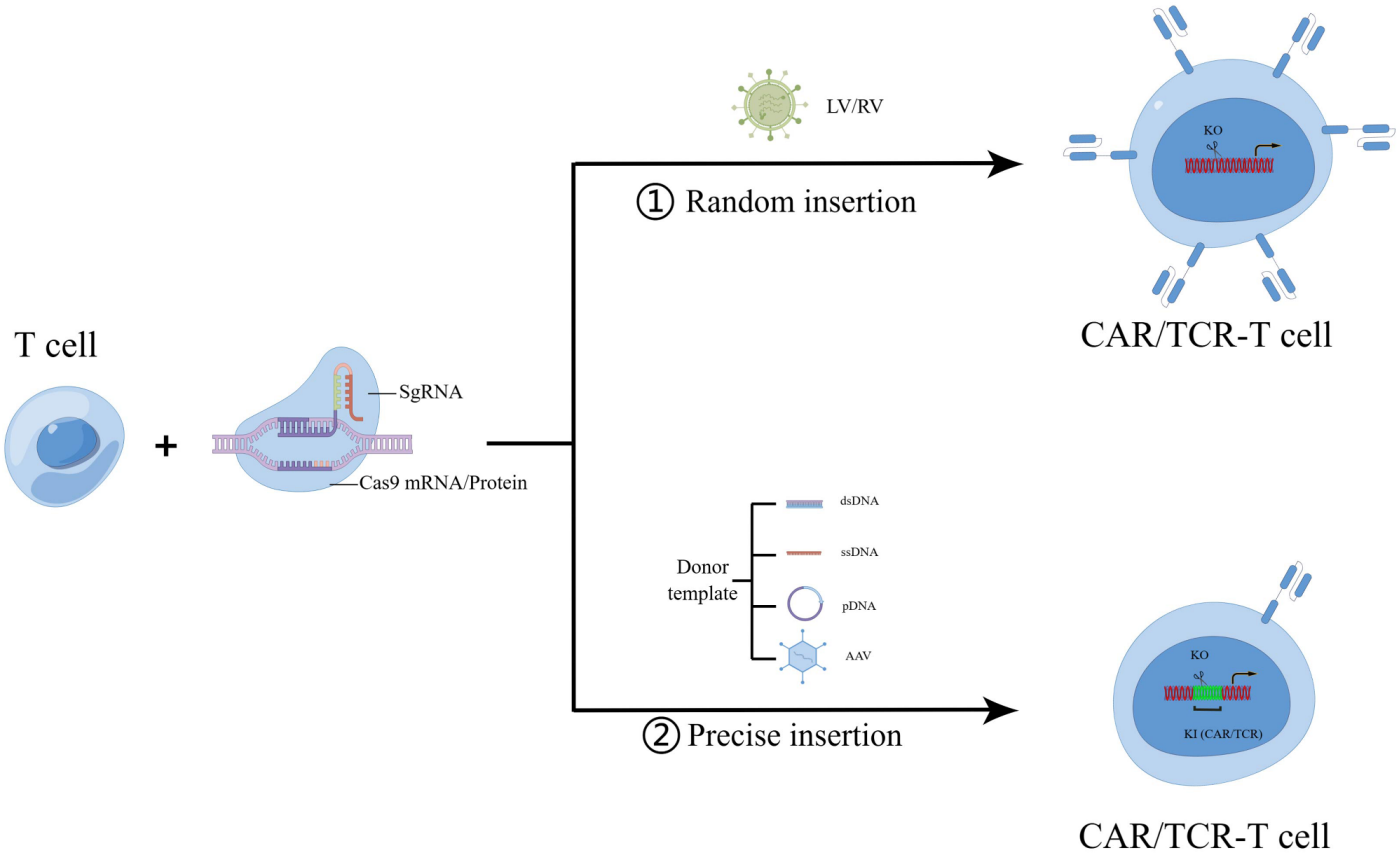
CRISPR mediate gene KO and KI strategy in CAR/TCR-T cell therapy. To achieve the formation of CAR/TCR-T cells, sgRNA and Cas9 protein are co-transposed into T cells, while CAR/TCR can enter T cells through two primary methods, eventually resulting in CAR/TCR-T cells. (1) Random insertion via LV/RV: The CAR or TCR is randomly inserted into T cells using LV or RV. (2) Precise insertion: This method facilitated by a donor template. Various forms of templates such as dsDNA, ssDNA, pDNA, or AAV are employed for site-specific integration of CAR or TCR into the T cells. sgRNA, single guide RNA; LV, lentivirus; RV, retrovirus; KO, knockout; KI, knockin; dsDNA, double strain DNA; ssDNA, single strain DNA; pDNA, plasmid DNA; AAV, Adeno-associated virus

Over the years, extensive research has been undertaken to deliver the CRISPR system into human primary T cells in three different forms: (i) Viral delivery of CRISPR vectors, such as LV or adeno-associated virus (AAV), (ii) Cas9 mRNA combined with synthetic guide RNA, (iii) Binding of Cas9 protein and synthetic guide RNA to form RNP complex [10, 11] (Fig. 1). The efficiency of gene knockout using CRISPR/Cas9 gene editing is relatively high, with the efficiency at the PD-1 locus exceeding 75%. Previous researchers have integrated guide RNA and CAR into a single vector, subsequently electro-transferring Cas9 mRNA to create gene-edited CAR-T cells. The efficacy of CRISPR/Cas9-mediated knockout hinges on the choice of target and guide RNA. To enhance knockout efficiency, researchers introduced MS (2’-Omethyl 3’-phosphorothioate) or MSP (2’-O-methyl 3’-thio PACE) modifications to the guide RNA. After binding the modified guide RNA to the Cas9 protein, they electrotransfected it into human primary T cells and CD34 + hematopoietic stem cells simultaneously. The results demonstrated a 2.4-fold increase in indel frequencies for MS-modified sgRNAs compared to unmodified ones (30.7% vs. 12.8%), significantly improving genome editing efficiency [12].

### Current research on CAR-T cell production using the CRISPR/Cas12a system

Cas12a (Cpf1) has two major isoforms, AsCpf1 and LbCpf1, known for higher specificity toward human cells compared to Cas9. It is now understood that Cas12a cleaves genomic DNA, generating sticky ends and displaying greater susceptibility to homologous recombination repair [13]. AAV6 vectors have been employed to engineer CrTRAC, crPDCD1, and CD19 CAR into one vector, achieving a simultaneous knockin efficiency of 37%, seven times that of the CRISPR/Cas9 system. The AAV-Cpf1 KIKO system established a precedent for the efficient expression of two CARs in the same T cell, facilitating the clinical application of bispecific CAR-T cells. Despite high gene knockin and knockout efficiency, Cas12a RNP cleavage efficiency was relatively low [14]. Researchers addressed this by developing a mutated version, AsCas12a Ultra, carrying M537R and F870L mutations. These mutations significantly enhanced knockout and knockin efficiency, especially in T cells, with single transgene knockin reaching up to 60% and double knockin up to 40% [15].

### Current research on CAR-T cell production using the CRISPR/Cas13d system

Tieu et al. introduced MEGA-CRISPR, a CRISPR/Cas13d-based tool [8], which utilizes tailored gRNA design to edit target RNA sequences with precision. This technology shows promise in enhancing CAR-T cell therapy efficacy by addressing T cell exhaustion and improving anti-cancer capabilities within the tumor microenvironment. Empirical validation in murine models demonstrates MEGA-CRISPR’s ability to enhance tumor cell killing efficiency, leading to significant tumor suppression and prolonged survival. Despite its potential, challenges such as complex technology and safety assessment hinder its widespread use. Further research and clinical validation are necessary to optimize MEGA-CRISPR for clinical applications.

### CRISPR/Cas system-mediated loci-specific knockin in CAR-T cells

To date, CAR-T cells have been primarily transduced using γ-retroviral vectors or lentiviral vectors. However, these methods result in random DNA integration in T cells and carry the risk of malignant transformation. To overcome this problem, one intriguing strategy is site-specific gene integration. By utilizing target-directed nucleases to create a double-strand break at a specific genomic locus, CAR transgenes can be integrated into the T cell genome via homologous recombination.

In 2017, Michel Sadelain’s group employed knockin techniques to insert the CD19 CAR gene into the TRAC locus, generating TRAC CAR-T cells. In comparison to CAR-T cells infected with retroviral vectors, the CD19 CAR knockin CAR-T cells exhibited diminished differentiation and depletion, while demonstrating significantly improved anti-tumor effects in mouse models [16]. In a melanoma mouse model, TCR-T cells generated with linear double-stranded DNA (dsDNA) as an HDR template exhibited more pronounced inhibition of melanoma growth compared to TCR-T cells generated with lentiviral vectors [10]. By utilizing non-viral, gene-specific targeted CAR-T cells through CRISPR-Cas9 at the PD-1 locus, it was demonstrated that non-viral, gene-specific integrated CAR-T cells offer both high safety and efficacy. This provides an innovative technology for CAR-T cell therapy of B-ALL [17]. A novel approach was devised to create targeted knockin CAR-T cells by employing modified plasmid DNA as a donor (referred to as pTRAC-CAR-T cells). In a murine leukemia model, the anti-tumor efficacy of these pTRAC-CAR-T cells was assessed and compared with CAR-T cells generated using AAV as the donor template. Results indicated that both variants of CAR-T cells demonstrated comparable anti-tumor effects. The well-established and cost-effective GMP (Good Manufacturing Practice) production of plasmid vectors highlights the feasibility of leveraging pTRAC-CAR-T cells generated from plasmid templates through the CRISPR/Cas9 system for prompt integration into future clinical trials [18].

In summary, there are presently three primary HDR template types for targeted gene delivery into primary T cells using the CRISPR system: (1) AAV-dependent target gene delivery [14, 16]; (2) linear ssDNA/dsDNA target gene delivery [10, 19]; and (3) plasmid DNA target gene delivery (Fig. 1). When employing AAV6 for target genes, one method involves co-transferring synthetic guide RNA and Cas9 mRNA via electrotransfer into human primary T cells, specifically targeting the TRAC site, followed by the introduction of AAV6 carrying the CAR gene. This approach achieves an editing efficiency of up to 45.6% [16]. An alternative strategy involves designing the guide RNA, purifying the Cas9 protein, creating an RNP complex in vitro, electrotransfecting it into T cells, and subsequently introducing AAV6 carrying the CAR gene. Using this method, the knockin efficiency can reach approximately 50% in T cells. In instances where linear dsDNA is utilized for knockin into the TRAC or PD-1 locus of human primary T cells, the knockin efficiency of the CAR/TCR gene is approximately 10-20% [10, 17]. Plasmid vectors, employed as HDR templates with the CRISPR/Cas9 system, necessitate the incorporation of a guide RNA sequence at each end of the homologous arm of the target gene, in addition to vectors containing guide RNA. The presence of the vector containing guide RNA significantly enhances the knockin efficiency, resulting in a 4-8-fold increase compared to the vector without guide RNA [18]. Both the advantage and disadvantage were showed in Table 2.

**Table 2:** CRISPR/Cas9-based CAR-T cells production and advantage/disadvantage

Cas9 format	CAR insertion	KI efficiency	Advantage	Disadvantage
Protein	LV/RV	depend on virus titer	stable and efficient	malignant transformation
mRNA	AAV	up to 50%	loci-specific	malignant transformation
Protein	AAV	up to 50%	loci-specific	malignant transformation
Protein	Linear dsDNA	about 20%	loci-specific	lower efficiency, GMP hard
Protein	pMini-CCS vector	no more than 20%	loci-specific, GMP easy	lower efficiency

## The application of CRISPR/Cas-based CAR-T cells

The utilization of CRISPR-based CAR-T cells encompasses several key facets, including the generation of universal CAR-T cells, overcoming immune checkpoint inhibition, and mitigating CAR-T cell fratricide. Subsequently, a detailed exploration of the application of CRISPR-based CAR-T cells in both scientific investigation and clinical settings will be provided.

### Generation of universal CAR-T cells

Currently, most CAR-T cell manufacturing relies on T cells sourced from autologous peripheral blood mononuclear cells (PBMCs). However, the costly and time-consuming production process may impede the accessibility of CAR-T cell therapy for individuals in urgent need, including those with rapidly progressing diseases or those unable to obtain potent autologous T cells due to inherent T cell defects [20]. In such scenarios, the utilization of off-the-shelf CAR-T products derived from healthy donors could potentially address these challenges. Nevertheless, the significant obstacle of acute and chronic graft-versus-host disease (GVHD) looms over this intriguing concept. To mitigate the risk of GVHD, CAR-T cells can be derived either from the patient’s previous HLA-matched hematopoietic stem cell transplant (HSCT) donor or through the genetic modification of CAR-T cells. Researchers have turned to gene editing technology to disrupt genes encoding the T cell receptor (TCR) and major histocompatibility complex (MHC), both of which contribute to alloreactivity. Two critical genes, TRAC and TRBC, encode endogenous TCR chains, with the TRAC locus serving as an ideal target for gene knockout and CAR knockin.

**Fig. 2:**
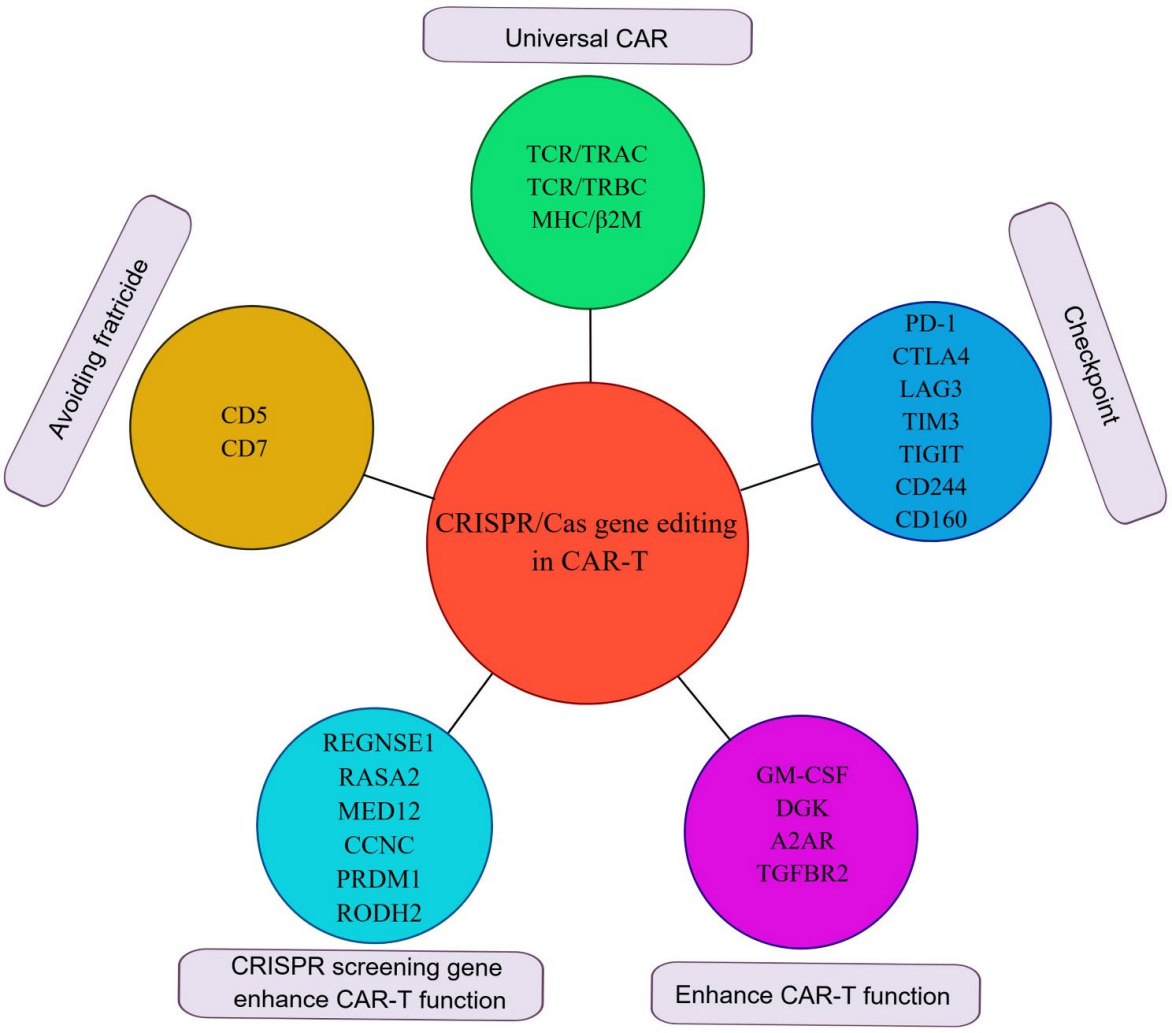
The gene editing site and application used by CRISPR system to enhance CAR-T cell function

In addition, Georgiadis et al. pioneered the creation of TCR-knockout CAR-T cells by integrating a self-inactivating lentiviral platform with the CRISPR/Cas system. Their study demonstrated that these TT CAR-T cells exhibit superior potency compared to TCR-positive CAR-T cells. Another promising approach to diminish the allogeneic response involves the ablation of MHC class I by targeting B2M. Researchers have successfully generated TRAC, B2M, and PDCD1 multiplex knockout CAR-T cells targeting CD19 or prostate stem cell antigen (PSCA), exhibiting reduced alloreactivity coupled with enhanced anti-tumor activity [21]. (Fig. 2)

### Disrupting immune checkpoint inhibitors

T cells express inhibitory receptors on their surface, contributing to T cell exhaustion, including PD-1, CTLA4, TIGIT, LAG-3, CD244, CD160, TIM3, and others. The suppressive tumor microenvironment and tumor cells can induce T cell anergy and exhaustion by upregulating inhibitory immune checkpoint signaling [22]. Repeated encounters with tumor cells lead CAR-T cells to adopt an exhausted phenotype primarily due to the upregulation of immune inhibitory receptors by tumor cells [22–24]. Knocking out these receptors enhances T cells’ ability to recognize tumor antigens. The PD-1/PD-L1 signaling pathway modulates T cell proliferation, activation, exhaustion, and immune tolerance [25]. Blocking the PD-1/PD-L1 axis on T cells has been documented to enhance CAR-T cell function [26, 27]. Inhibiting the expression of immunosuppressive receptors like PD-1 has been extensively studied in hematologic and solid tumors. Current evidence suggests that PD-1 knockout activates the T cell immune response against tumors, particularly in lung cancer. Additionally, PD-1 knockout has demonstrated increased anti-tumor activity in CD19 CAR-T cells for hematological malignancies, GPC3 CAR-T cells for liver cancer, and mesothelin CAR-T cells for human ductal adenocarcinoma. Knocking out molecular markers associated with T cell exhaustion, like PD-1 and CTLA4, in Universal CARs improved their tumor-killing activity. Taken together, the above findings suggest that CAR-T cell therapies designed based on immune checkpoints offer potential advantages in controlling solid tumors, presenting a novel strategy for adoptive T cell therapies.

Combining immunotherapy with CAR-T cells and immune checkpoint blockade has shown tumor regression. However, systematic administration of immune checkpoint/ligand monoclonal antibodies poses a risk of immune-related adverse events (IRAEs) [28]. Genetically disrupting intrinsic PD-1 signaling using CRISPR/Cas9 can minimize toxicity while preserving CAR-T cells’ effector function. Extensive evidence supports the idea that abrogating PD-1 with CRISPR/Cas9 enhances the anti-tumor potency of both allogeneic and autologous CAR-T cells in hematological malignancies and solid tumors during preclinical and clinical evaluations [11, 29].

To counterbalance the negative impact of the Fas/FasL axis on T cell survival, Ren et al. developed a practical one-shot CRISPR system. They incorporated multiple gRNAs into a lentiviral vector along with a CAR transgene, resulting in the generation of Fas-resistant universal CAR-T cells and PD-1/CTLA-4 dual-resistant universal CAR-T cells. Despite a decrease in knockout efficacy with an increased number of targeted genes, Fas-deficient CAR-T cells exhibited enhanced resistance to AICD, leading to prolonged persistence [30]. This finding was supported by Zhang et al., who reported robust efficacy of LAG-3-deficient CAR-T cells in a preclinical model [31]. (Fig. 2)

### Avoiding fratricide in CAR-T cell therapy targeting T cell malignancy

While CAR-T cell therapy has demonstrated remarkable success in treating advanced B-cell malignancies and adult relapsed/refractory multiple myeloma, its effectiveness is currently limited, and treatment options for refractory and relapsed T cell-related tumors remain scarce. A significant challenge in CAR-T cell therapy lies in the presence of targeted T cell-pan markers on CAR-T cells, potentially resulting in self-activation, fratricide, and impaired functionality of CAR-T cells. These factors significantly impact the efficacy of CAR-T cell therapy for T cell-related tumors.

CD5 and CD7 are transmembrane proteins that are highly expressed in T cell malignancies [32, 33], with restricted expression mainly to T cells, NK cells, and B1 cells, making them attractive targets for CAR-T cell therapy. However, the presence of shared antigens on tumor cells and CAR-T cells could lead to fratricide. To mitigate this issue, researchers have explored genetic editing of the CD5 and CD7 genes in CAR-T cells using the CRISPR/Cas9 system [34].

In the development of CD5-targeted CAR-T cells for T cell malignancies, researchers encountered a challenge of self-mutilation during the in vitro generation process. To address this, they employed the CRISPR/Cas9 system to knockout CD5 on CAR-T cells. This approach resulted in reduced levels of CAR-T cell activation while significantly increasing the expression of CD5 CARs [35]. In an experiment conducted by the Carl June group, CD5 knockout CAR-T cells injected into a mouse Jurkat T tumor model led to a significant extension of the mice’s survival. Another target, CD7, exhibited high expression not only in T lymphoma cells but also in normal T cells. Knocking out CD7 using the CRISPR/Cas9 system did not impact T cell proliferation or killing ability. In an AML mouse model, tumors largely disappeared when mice were injected with CD7-knockout CAR-T cells. TCR-, β2M-, and CD7-knockout universal CAR-T cell therapy has been investigated in clinical trials for treating T cell acute lymphoblastic leukemia (T-ALL). (Fig. 2)

### CRISPR/Cas9-based gene-knockout enhances CAR-T cell function

T cells express a variety of inhibitory receptors on their surface, including PD-1, CTLA4, TIGIT, LAG-3, CD244, CD160, and TIM3, contributing to T cell exhaustion [22, 23, 36]. Knocking out these receptors has been shown to enhance T cells’ ability to recognize tumor antigens. In this respect, the suppression of PD-1 expression has been extensively studied in hematological and solid tumors [25]. PD-1 knockout activates the T cell immune response against tumors, providing a potential treatment for lung cancer [37]. Moreover, PD-1 knockout enhances the anti-tumor activity of CD19 CAR-T cells in hematological malignancies. To address the negative effect of the Fas/FasL axis on T cell survival, Ren et al. developed a practical one-shot CRISPR system. Multiple gRNAs were incorporated into a lentiviral vector along with the CAR transgene, resulting in the generation of Fas-resistant universal CAR-T cells and PD-1/CTLA-4 dual-resistant universal CAR-T cells. Despite a decrease in knockout efficacy with an increasing number of targeted genes, Fas-deficient CAR-T cells demonstrated greater resistance to AICD and prolonged persistence [30]. Similarly, Zhang et al. reported robust efficacy of LAG-3-deficient CAR-T cells in preclinical models [31].

Beyond immune checkpoints, there are other molecules whose knockout can improve CAR-T cell function or reduce side effects in therapy. Fas, a member of tumor necrosis factor-alpha (TNF-α), mediates cell death through the Fas-FasL signaling-induced activation-induced cell death (AICD), potentially reducing CAR-T cell activation. Producing anti-Fas CAR-T cells using the CRISPR/Cas9 system can improve CAR-T cell tolerance to AICD and prolong the survival of tumor-bearing mice. TGF-β, binding to the TGF-β receptor (TGFBRI) on the T cell membrane, activates downstream signaling pathways SMAD2 and SMAD3, leading to reduced cytokine production and increased cytotoxicity [38]. Knocking out TGF receptor II (TGFBR2) in CAR-T cells using the CRISPR-Cas9 system promotes the differentiation of CAR-T cells into central memory and effector cells, enhancing tumor clearance in solid tumor models [39].

It has been established that adenosine, an immunosuppressive factor, activates the adenosine A2A receptor, inhibiting the activation of multiple immune cells and suppressing the anti-tumor immune response. Using CRISPR/Cas9 to knock out the adenosine A2A receptor was found to enhance the anti-tumor effects of Her2-targeted CAR-T cells in breast cancer [40]. Glycerol diglyceride kinase (DGK) metabolizes glycerol diesters into phosphatidic acid. Knocking out DGK enhances TCR signaling, increasing the killing capacity of T cells in glioma [41]. Granulocyte macrophage colony-stimulating factor (GM-CSF), mainly produced by T cells and macrophages, has been targeted in clinical trials involving leukemia patients. GM-CSF knockout, coupled with the generation of CAR-T cells targeting IL6, has been shown to reduce autocrine production of IL-1 and IL-6, subsequently decreasing cytokine release syndrome (CRS) in patients. GM-CSF knockout has also been found to enhance the anti-tumor efficacy of CD19-targeting CAR-T cells in mice, prolonging the survival time of tumor-bearing mice [42, 43]. (Fig. 2)

### Gain of CAR-T cell function by CRISPR screening

**Fig. 3:**
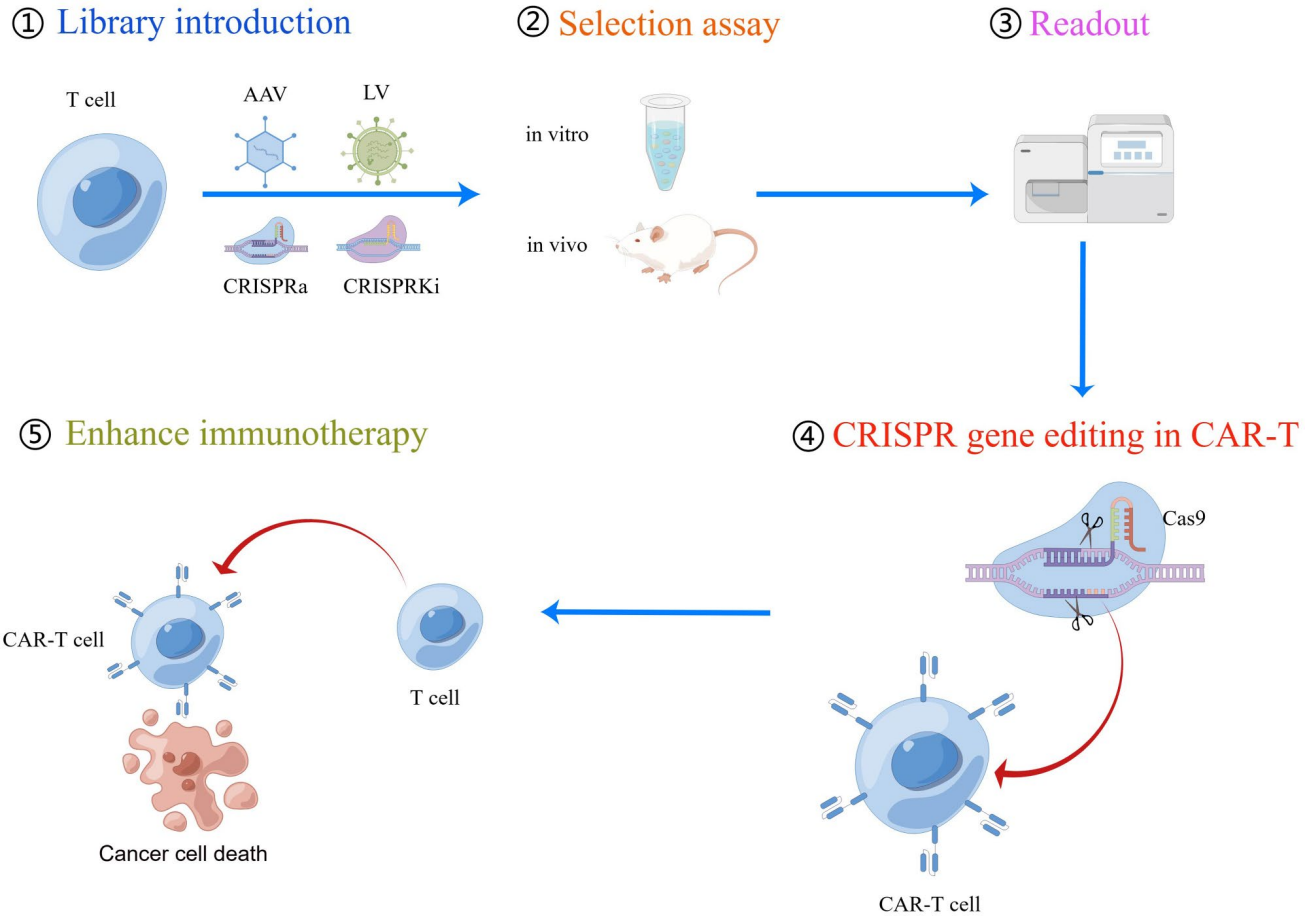
The flow chart of CRISPR screening process to screen genes to enhance CAR-T cell function. (1) CRISPRa or CRISPRi components (including guide RNA library) can be delivered into T cells using viral vectors such as AAV or lentivirus. (2) Depending on the phenotypes of interest, either in vitro or in vivo assays can be utilized for guide RNA selection. (3) Next-generation sequencing is then conducted to assess guide RNA enrichment or deletion. (4) Target gene editing is performed in T cells using CRISPR/Cas technology. (5) The ultimate objective of these screens is to evaluate the capacity to enhance the recognition and killing of tumor cells by CAR-T cells. CRISPRa, CRISPR activation; CRISPRi, CRISPR interference; AAV, Adeno-associated virus

Zhang Feng’s research group has advanced CRISPRa and CRISPRi technologies, increasingly employed for screening and modifying genes related to T cell function [44–46]. These technologies entail the introduction of CRISPRa and CRISPRi libraries into T cells using AAV or LV vectors. Through in vitro and in vivo experiments, target genes associated with T cell cytotoxicity are identified, enhancing the tumor-killing ability of CAR-T cells through single or multiple gene editing. This innovative technology opens new possibilities for T cell therapy (Fig. 3).

Sidi Chen has developed a hybrid genetic screening system in which Sleeping Beauty (SB) transposons and a sgRNA cassette are nested in adeno-associated virus (AAV) [47]. This system enables efficient gene editing in primary murine T cells and provides a screening readout. In vivo, AAV–SB-CRISPR screens were conducted to identify membrane protein targets in CD8 + T cells in mouse models of glioblastoma (GBM). The screen hits, including PDIA3, MGAT5, EMP1, and LAG3 gene editing, were validated through the adoptive transfer of CD8 + T cells, enhancing the survival of GBM-bearing mice in both syngeneic and T cell receptor transgenic models [47]. In another study by Hongbo Chi et al., an in vivo pooled CRISPR-Cas9 screening system was employed to target REGNASE-1 in CD8 + T cells. The results demonstrated that CD8 + T cells with REGNASE-1 knockout exhibited long-lived effector cells with extensive accumulation, better persistence, and robust effector function in tumors [48]. Further research showed that knockout of REGNASE-1 enhances CAR-T cell persistence and CAR-T-mediated antitumor immunity in murine and human xenograft B-ALL models. This was achieved by targeting TCF7 mRNA to inhibit the formation of precursors of exhausted T cells (T_PEX_) [49].

Alexander Marson conducted multiple genome-wide CRISPR knock-out screens under different immunosuppressive conditions to identify genes that can be targeted to prevent T cell dysfunction. These screens revealed that RASA2, a RAS GTPase-activating protein (RasGAP), serves as a signaling checkpoint. It has been established that RASA2 is downregulated upon acute T cell receptor stimulation and gradually increases with chronic antigen exposure. Ablation of RASA2 was found to enhance MAPK signaling and CAR-T cell cytolytic activity in response to the target antigen [50]. CRISPR screening also identified that inactivating MED12 or CCNC in CAR-T cells increased T cell expansion and metabolic fitness, ultimately enhancing T cell effector activity [51].

Jeremy N. Rich et al. utilized CRISPR screening in CAR-T cells to identify the knockout of TLE4 and IKZF2, which enhanced the efficacy of CAR-T cells against glioblastoma [52]. Douglas R. Green et al. employed CRISPR screening to identify inhibitors of antigen-specific memory T cell generation in vivo. Their study revealed the crucial role of the cBAF complex in T cell fate decisions and CAR-T cells [53]. Sidi Chen’s lab devised a dead-guide RNA (dgRNA)-based CRISPR activation screen system in primary CD8 + T cells. Through this system, they identified gain-of-function targets for CAR-T engineering. They demonstrated that overexpressing PRODH2, which takes part in proline metabolism, enhances CAR-T-based killing and in vivo efficacy in various cancer models. These findings not only present a method for identifying immune boosters with gain-of-function, but also highlight PRODH2 as a target to enhance CAR-T efficacy by reshaping gene expression and metabolic programs [54].

Sidi Chen developed a system called CLASH that harnesses Cas12a/Cpf1 mRNA and pooled adeno-associated viruses to facilitate simultaneous gene editing and precise transgene knockin using massively parallel homology-directed repair. This system generates a pool of stably integrated mutant variants, each with targeted gene editing. They applied this technology in primary human T cells and observed that mutation of PRDM1 in CAR-T cells resulted in increased proliferation, stem-like properties, central memory, and longevity. Consequently, these cells demonstrated higher efficacy in vivo in CD19 + CD22 + NALM6 cancer models and in the HER2 + HT29 tumor model [55]. (Fig. 3)

### The clinical trial for CRISPR-based CAR-T cells

In a Phase I study, CTA101, universal CD19/CD22-targeting CAR-T cells, were infused into patients with r/r ALL. These CAR-T cells featured a CRISPR/Cas9-disrupted TRAC region and CD52 gene to prevent host immune-mediated rejection. On day 28 post-infusion, the complete remission (CR) rate was 83.3%. With a median follow-up of 4.3 months, these CRISPR/Cas9-engineered CAR-T cells displayed a manageable safety profile and significant antileukemia activity. The technology achieved highly efficient, high-fidelity gene editing, resulting in the production of universal CAR-T cells without observable genotoxicity or chromosomal translocations [56].

Utilizing next-generation CRISPR/Cas9 editing, CAR expression was linked to multiplexed DNA editing of TRAC and CD52 by incorporating self-duplicating CRISPR guide RNA expression cassettes within the 3’ long terminal repeat of a CAR19 lentiviral vector. In a study treating children with relapsed/refractory CD19-positive B cell acute lymphoblastic leukemia (B-ALL), six patients received TT52CAR19 T cells. Four of these patients exhibited cell expansion, achieved flow cytometric remission, and subsequently underwent allogeneic stem cell transplantation. While two patients experienced grade II cytokine release syndrome requiring intervention, one patient developed transient grade IV neurotoxicity, and another developed skin GVHD, resolving after transplant conditioning. This study demonstrated the feasibility, safety, and therapeutic potential of CRISPR-engineered immunotherapy [57].

Furthermore, TCR and B2M double-disrupted universal CAR T cells were generated from healthy donor T cells using lentivirus and CRISPR/Cas9 genome-editing technology to treat DLBCL. Although the study had limitations regarding safety and clinical response, the pooled analysis represents a significant advancement in the development of universal CAR T cells for improving safety, efficacy, and feasibility in patients with hematological malignancies [58]. 

In a single-arm phase I dose-escalation clinical trial evaluating PD1-19bbz in adult patients with r/r B-NHL, twenty-one patients received PD1-19bbz infusion. Most patients had advanced disease stages and intermediate or worse risk stratifications. Notably, some participants exhibited high levels of programmed death ligand-1 (PD-L1) expression in pre-treatment tumor samples. PD1-19bbz demonstrated promising efficacy with a manageable toxicity profile in this first-in-human study of non-viral specifically integrated CAR-T products. A phase I/II trial of PD1-19bbz in a larger patient cohort is currently underway [17]. 

PD-1-mediated immunosuppression likely limits the efficacy of CAR-T cells in solid tumors. Researchers utilized CRISPR/Cas9 to create PD-1 and TCR deficient mesothelin-specific CAR-T (MPTK-CAR-T) cells and assessed them in a dose-escalation study with 15 patients. No dose-limiting toxicity or unexpected adverse events occurred. Only two patients showed stable disease as the best overall response. Circulating MPTK-CAR-T cells peaked at days 7–14 and declined thereafter. TCR-positive CAR-T cells were predominantly detected post-infusion. Animal models also confirmed the reduced persistence of TCR-deficient CAR-T cells. These findings establish the feasibility and safety of CRISPR-engineered CAR-T cells with PD-1 disruption and underscore the role of natural TCR in CAR-T cell persistence in solid tumor therapy [59].

CAR-T therapy for T cell malignancies faces challenges such as CAR-T cell fratricide and blast contamination. Allogeneic CAR-T cells from healthy donors offer blast-free products but risk graft-versus-host disease (GvHD) and rejection. We developed CD7-targeting CAR-T cells (RD13-01) from healthy donors with genetic modifications for fratricide resistance, GvHD prevention, and enhanced antitumor function. In a phase I trial (NCT04538599) with twelve patients (eleven with T cell leukemia/lymphoma, one with CD7-expressing AML), all met endpoints, with eleven proceeding to efficacy evaluation. No dose-limiting toxicity, GvHD, neurotoxicity, or severe cytokine release syndrome (grade ≥ 3) occurred. At 28 days post-infusion, 81.8% showed objective responses, with a complete response rate of 63.6% (including the AML patient). Three patients underwent allogeneic stem cell transplantation, and four remained in complete remission at a median follow-up of 10.5 months. CMV/EBV reactivation was observed, and one patient died from EBV-associated DLBCL. Expansion of CD7-negative T cells was detected post-infusion. This Phase I trial demonstrates the safety and efficacy of RD13-01 allogeneic CAR-T cells for CD7 + tumors [60].

In Table 3, we summarize the clinical trials of CRISPR- based CAR-T therapies. The clinical trials demonstrate the potential of CRISPR-based CAR-T cell therapy in treating hematological malignancies and solid tumors, but it also presents several challenges in terms of safety and effectiveness.

**Table 3:** Clinical trials of CRISPR-based CAR-T cell therapy

Target-antigen	Characteristics	Application	Therapeutic efficacy	Clinical trials
CD19	PD1 integration	B-NHL	87.5% CR	NCT04213469
CD19/CD22	TRAC and CD52 disrupted	B-ALL	60% (3/5) CR/CRi	NCT04227015
Mesothelin	PD-1 and TCR disrupted	Mesothelin-positive solid tumors	13.3% (2/15) stable disease	NCT03545815
CD19	TRAC and CD52 disrupted	B-ALL	4/6 patients proceeded to HSCT	NCT04557436
CD7	TRBC1, TRBC2, CD52 and CD7 disrupted	T-ALL	2/3 patients proceeded to HSCT	ISRCTN15323014
CD7	TRAC, CD7, and RFX5 disrupted	T-cell leukemia/lymphoma, CD7-expressing AML	63.6% (7/11) CR	NCT04538599

## Conclusion and prospects

The development of the CRISPR system used in human primary T cells has undergone rapid progression over the past decade, especially for gene knockout applications, with relatively high efficiency for both single and multiple gene targeting. Knockout of molecules involved in T cell exhaustion and suppression of T cell function by the CRISPR system can significantly enhance the function of CAR-T cells, providing a new approach for CAR-T cell applications in solid tumors and hematological malignancies.

While CRISPR-based CAR-T cell therapy presents great promise, its application in preclinical studies or clinical trials is fraught with challenges, particularly concerning safety and efficacy. Here, we explore the pivotal safety considerations associated with CRISPR technology and propose potential solutions. (1) Mitigation of Off-Target Effects: The CRISPR system can sometimes induce unwanted mutations at off-target sites within the genome. By employing advanced bioinformatics tools for designing gRNAs and utilizing CRISPR variants with enhanced specificity, the occurrence of off-target effects can be controlled. (2) Immune Response and Immunogenicity: CRISPR-edited cells may trigger immune responses in recipients, potentially leading to rejection or adverse reactions. Strategies to reduce immunogenicity include the selection of non-immunogenic CRISPR components or the use of immunomodulatory agents, which are currently under investigation. (3) Insertional Mutagenesis: Viral vectors used in CRISPR delivery could integrate the CAR gene into the host genome, thereby posing risks of insertional mutagenesis and oncogenesis. Employing non-integrating delivery methods such as mRNA-based or PiggyBac transposon-based approaches can mitigate these risks. (4) Genomic Stability: Ensuring the genomic stability of CRISPR-edited cells is crucial to prevent unintended genetic alterations. Periodic genomic profiling and long-term monitoring of treated patients are essential to assess the stability of edited genomes and to detect any potential abnormalities. (5) Long-Term Effects: Long-term follow-up studies are necessary to evaluate the durability of the therapeutic response and monitor any late-onset adverse events associated with CRISPR-based CAR-T cell therapies. This includes assessing potential long-term effects on the immune system, hematopoiesis, and overall health. Addressing these safety concerns requires rigorous preclinical evaluation, careful patient selection, and continuous monitoring during clinical trials. Another challenge lies in ensuring the efficacy of the treatment, including stable expression of CAR-T cells in the body and their ability to recognize and eliminate tumor cells. This necessitates consideration of the complexities of cellular engineering in the design and optimization of therapeutic protocols and appropriate evaluation and adjustments during clinical trials. Furthermore, the cost and complexity of manufacturing CRISPR-based CAR-T cell therapies pose additional challenges. Optimizing production processes, enhancing the standardization of technology, and reducing manufacturing costs are key factors in advancing this field. In summary, CRISPR-based CAR-T cell therapies hold immense potential in the treatment of various diseases, particularly cancer. While CRISPR/Cas9 remains the most explored system due to its efficiency and relatively better understood characteristics, both CRISPR/Cas12a and CRISPR/Cas13d show promise, each with their unique advantages. The safety and efficacy of these therapies are being actively investigated, and with further research, they can be optimized to provide safer and more effective treatments in the future.

## Data Availability

No datasets were generated or analysed during the current study.

## Abbreviations

CAR-T: Chimeric Antigen Receptor T cell
scFv: Single-Chain Fragment Variable
B-ALL: B-cell Acute Lymphoblastic Leukemia
NHL: Non-Hodgkin Lymphoma
MM: Multiple Myeloma
LV: Lenti-Virus
RV: Retroviral Vector
AAV: Adeno-Associated Virus
TALENs: Transcription Activator-Like Effector Nucleases
ZFNs: Zinc-Finger Nucleases
CRISPR: Clustered Regularly Interspaced Short Palindromic Repeats
DSB: Double-Stranded Break
HDR: Homology-Directed Repair
NHEJ: Non-Homologous End Joining
sgRNA: Single Guide RNA
PAM: Protospacer Adjacent Motif
CRISPRa: CRISPR Activation
CRISPRi: CRISPR Interference
PBMCs: Peripheral Blood Mononuclear Cells
GVHD: Graft-Versus-Host Disease
HSCT: Hematopoietic Stem Cell Transplant
TCR: T Cell Receptor
MHC: Major Histocompatibility Complex
PSCA: Prostate Stem Cell Antigen
IRAEs: Immune-Related Adverse Events
T-ALL: T cell Acute Lymphoblastic Leukemia
TNF-α: Tumor Necrosis Factor Alpha
AICD: Activation-Induced Cell Death
TGFBRI: TGF-β Binds to the TGF-β Receptor
TGFBR2: TGF Receptor II
DGK: Glycerol Diglyceride Kinase
GM-CSF: Granulocyte Macrophage Colony Stimulating Factor
CRS: Cytokine Release Syndrome
dsDNA: Double-Stranded DNA
SB: Sleeping Beauty
GBM: Glioblastoma
TPEX: Precursor Exhausted T Cells
RasGAP: RAS GTPase-Activating Protein
dgRNA: Dead-Guide RNA
OMEGA: Obligate Mobile Element-Guided Activity Omega
GMP: Good Manufacturing Practice
