# Correction: CRISPR/Cas-based CAR-T cells: production and application

**DOI:** 10.1186/s40364-024-00616-7

**Published:** 2024-07-23

**Authors:** Ping Song, Qiqi Zhang, Zhiyong Xu, Yueli Shi, Ruirui Jing, Dingcun Luo

**Affiliations:** 1 Department of Surgical Oncology, Affiliated Hangzhou First People’s Hospital, Westlake University School of Medicine, No. 261, Huansha Road, Shangcheng district, Hangzhou, 310006 Zhejiang P. R. China; 2 Bone Marrow Transplantation Center, the First Affiliated Hospital, Zhejiang University School of Medicine, Hangzhou, China; 3 Department of Respiratory Medicine, The Fourth Affiliated Hospital, International Institutes of Medicine, Zhejiang University School of Medicine, Yiwu City, China; 4 The Fourth Clinical Medical College, Zhejiang Chinese Medical University, Hangzhou, 310006 Zhejiang China

**Correction: Biomarker Research (2024) 12:54**

10.1186/s40364-024-00602-z

The authors wish to note Ruirui Jing as a co-Corresponding Author of the original article; the attribution of which was inadvertently missed during production of the manuscript.

